# Single cell and spatial transcriptomics analysis of kidney double negative T lymphocytes in normal and ischemic mouse kidneys

**DOI:** 10.1038/s41598-023-48213-2

**Published:** 2023-11-28

**Authors:** Sepideh Gharaie, Kyungho Lee, Kathleen Noller, Emily K. Lo, Brendan Miller, Hyun Jun Jung, Andrea M. Newman-Rivera, Johanna T. Kurzhagen, Nirmish Singla, Paul A. Welling, Jean Fan, Patrick Cahan, Sanjeev Noel, Hamid Rabb

**Affiliations:** 1 Department of Medicine, Johns Hopkins University, School of Medicine, Ross 965, 720 Rutland Ave, Baltimore, MD 21205 USA; 2 Department of Biomedical Engineering, Johns Hopkins University, School of Medicine, Baltimore, MD 21205 USA; 3 Department of Urology, Johns Hopkins University, School of Medicine, Baltimore, MD 21205 USA; 4 Department of Physiology, Johns Hopkins University, School of Medicine, Baltimore, MD 21205 USA

**Keywords:** Computational biology and bioinformatics, Immunology, Nephrology

## Abstract

T cells are important in the pathogenesis of acute kidney injury (AKI), and TCR^+^CD4^-^CD8^-^ (double negative-DN) are T cells that have regulatory properties. However, there is limited information on DN T cells compared to traditional CD4^+^ and CD8^+^ cells. To elucidate the molecular signature and spatial dynamics of DN T cells during AKI, we performed single-cell RNA sequencing (scRNA-seq) on sorted murine DN, CD4^+^, and CD8^+^ cells combined with spatial transcriptomic profiling of normal and post AKI mouse kidneys. scRNA-seq revealed distinct transcriptional profiles for DN, CD4^+^, and CD8^+^ T cells of mouse kidneys with enrichment of *Kcnq5*, *Klrb1c*, *Fcer1g*, and *Klre1* expression in DN T cells compared to CD4^+^ and CD8^+^ T cells in normal kidney tissue. We validated the expression of these four genes in mouse kidney DN, CD4^+^ and CD8^+^ T cells using RT-PCR and *Kcnq5*, *Klrb1*, and *Fcer1g* genes with the NIH human kidney precision medicine project (KPMP). Spatial transcriptomics in normal and ischemic mouse kidney tissue showed a localized cluster of T cells in the outer medulla expressing DN T cell genes including *Fcer1g*. These results provide a template for future studies in DN T as well as CD4^+^ and CD8^+^ cells in normal and diseased kidneys.

## Introduction

Acute kidney injury (AKI) is common in native kidneys leading to significant mortality and morbidity, while in kidney transplants AKI increases the length of hospital stay and reduces long- term allograft function^1,2^. Ischemia reperfusion (IR) injury and nephrotoxins are major causes of AKI. Pathogenesis of AKI is complex and involves several mechanisms including inflammation, apoptosis, tubular necrosis, production of reactive oxygen species, and epigenetic alterations^3,4^. White blood cells including neutrophils, dendritic cells, macrophages, B and T cells have been shown to mediate AKI. Analysis of kidney T cells has mainly concentrated on conventional CD4^+^ and regulatory CD4^+^CD25^+^FoxP3 Tregs during AKI^5,6^. However, renal double negative (DN) T cells are a unique subset of T cell receptor (TCR) αβ T lymphocytes, which do not express CD4^+^ and CD8^+^ cell markers^7–10^. DN T cell frequency in a normal human kidney can range between 18 and 61% of all renal T cells^7^, while the mouse kidney contains approximately 25% of DN T cells, which rapidly expand within 24 h after AKI^8^ and decrease 72 h post IR compared to sham operated mice^11^.

Kidney DN T cells have in vitro suppressive function against CD4^+^ T cells that protects the kidney against AKI and lung against IRI^11,12^. However, little is known about the molecular pathways engaged in DN during AKI. Although recent single cell studies have focused on mapping the landscape of CD4^+^ and CD8^+^ T lymphocytes in the kidney tissue^13–15^, the transcriptional and spatial landscapes of kidney-resident DN T cells in normal and post-ischemic kidney are relatively unknown.

Single-cell RNA sequencing (scRNA-seq) technology can provide insights into the abundance and functional state of different cell types as well as characterizations of their transcriptomes^16^. Spatial transcriptomics (ST) is a technique that uncovers transcriptional signatures within the spatial context of intact tissue by integrating histology with RNA-seq thereby enabling the mapping of transcriptional changes during AKI and repair^17,18^. We therefore performed and integrated spatial and single-cell transcriptomics to localize kidney T lymphocytes in murine normal and ischemic kidneys with a focus on DN T cells. By integrating scRNA-seq and spatial transcriptomics in normal and ischemic mouse kidney tissue, we identified a localized cluster of T cells expressing DN T cell genes including Fc Epsilon Receptor Ig (*Fcer1g*). This predominated in the outer medulla, a well-established region for leukocyte congestion during AKI. We also explored expression of our genes of interest in the NIH human kidney precision medicine project (KPMP) database.

## Results

### Single-cell transcriptomic analysis of T lymphocyte cell subtypes

**Figure 1:**
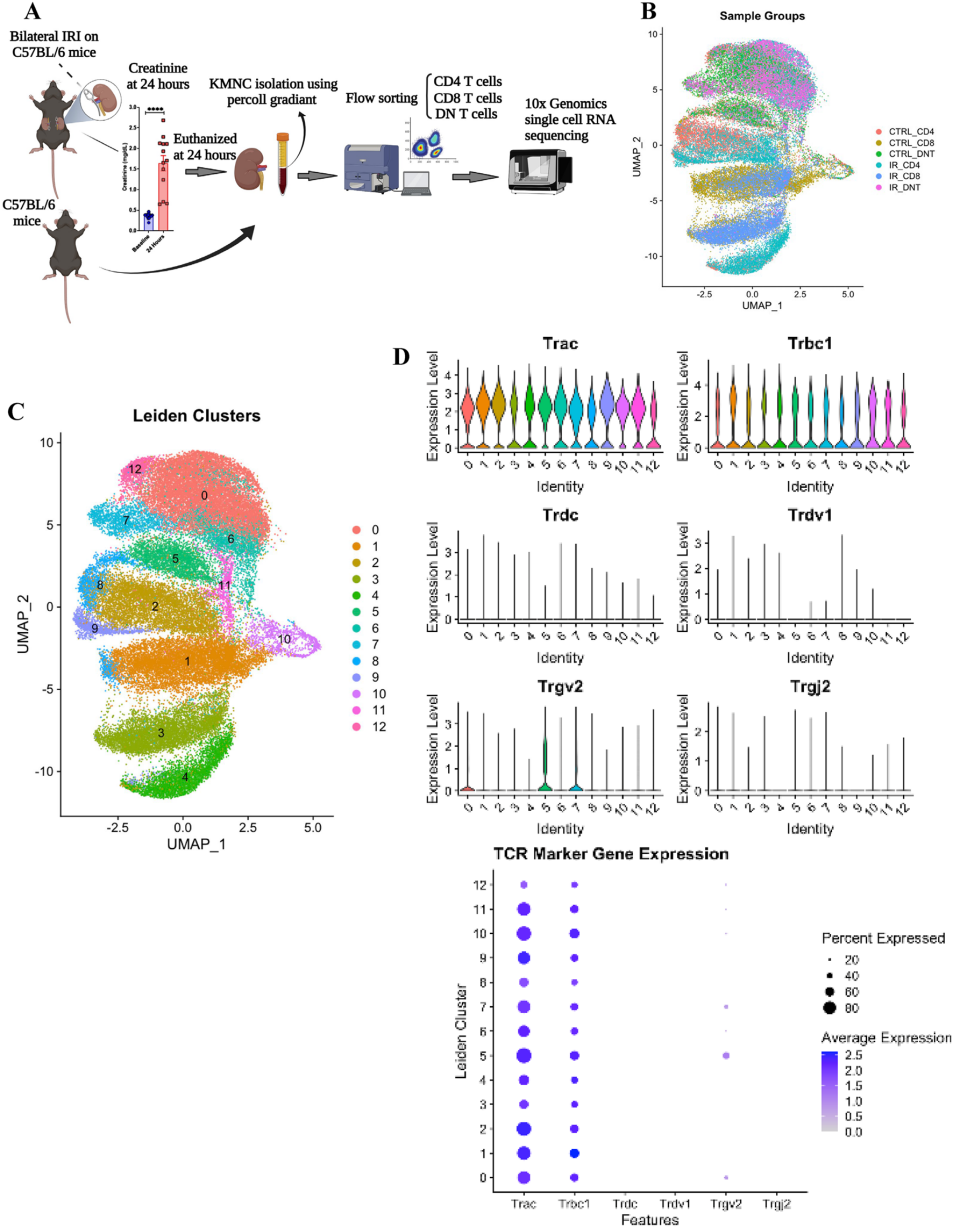
Single-cell transcriptomic analysis of T lymphocyte cells in murine normal and ischemic kidneys. (**A**) Experimental design and serum creatinine at the baseline and 24 h after bilateral ischemia reperfusion (IR) injury. (**B**) Uniform manifold approximation and projection (UMAP) graph from six cell types: control (ctrl) CD4^+^, ctrl- CD8^+^, ctrl- DN cells, IR- CD4^+^, IR- CD8^+^, and IR- DN T cells. (**C**) Detection of expression of T cell receptor (TCR)αβ^+^ in all clusters using Trac and Trbc1 markers. (**D**) Detection of expression of TCRδ and TCRγ in some clusters using Trdc, Trdv1, Tcrg-c, Trgj2, and Trgv2 markers.

We used an established surgical model of ischemic AKI in mice with 30 min of bilateral ischemia followed by reperfusion^19^. There was a significant elevation in serum creatinine at 24 h confirming ischemic AKI (Fig. 1A). To define global transcriptomic signatures of distinct T-lymphocyte populations, we performed scRNA-seq analysis of flow sorted CD45^+^ α/ß-TCR^+^ T cells from normal (control group) and ischemic (IR group) murine kidneys which were further sorted into 3 subpopulations: CD4^+^, CD8^+^ and DN (CD4^-^/CD8^-^) T cells. We then performed scRNA-seq library preparation on approximately 5000–10,000 cells per sample group using the 10X Genomics platform, a high-throughput and parallel droplet-based scRNA-seq platform (Fig. 1A). Next, we performed quality control to exclude potential doublets and low-quality libraries, defining doublets as cells with the top 5% of total reads per capture in accordance with the estimated doublet rate of the 10× Genomics platform^20^. Low-quality libraries expressed fewer than 500 detectable features or expressed mitochondrial features in greater than 20% of their total transcriptome. Filtering of features was also performed, where features expressed in fewer than 5 cells, mitochondrial and ribosomal features, and *Malat1* were removed. Next, batch correction was performed in Harmony in R to obtain adjusted embeddings without altering gene expression values^21^. Following quality control and batch correction, we obtained a filtered counts matrix with 19,378 features and 50,760 cells including CD4^+^, CD8^+^, and DN normal (control) with 9747, 9805, and 9349 cells respectively as well as CD4^+^, CD8^+^, and DN IR (ischemic) with 10,724, 6777, and 4358 cells respectively.

**Figure 2:**
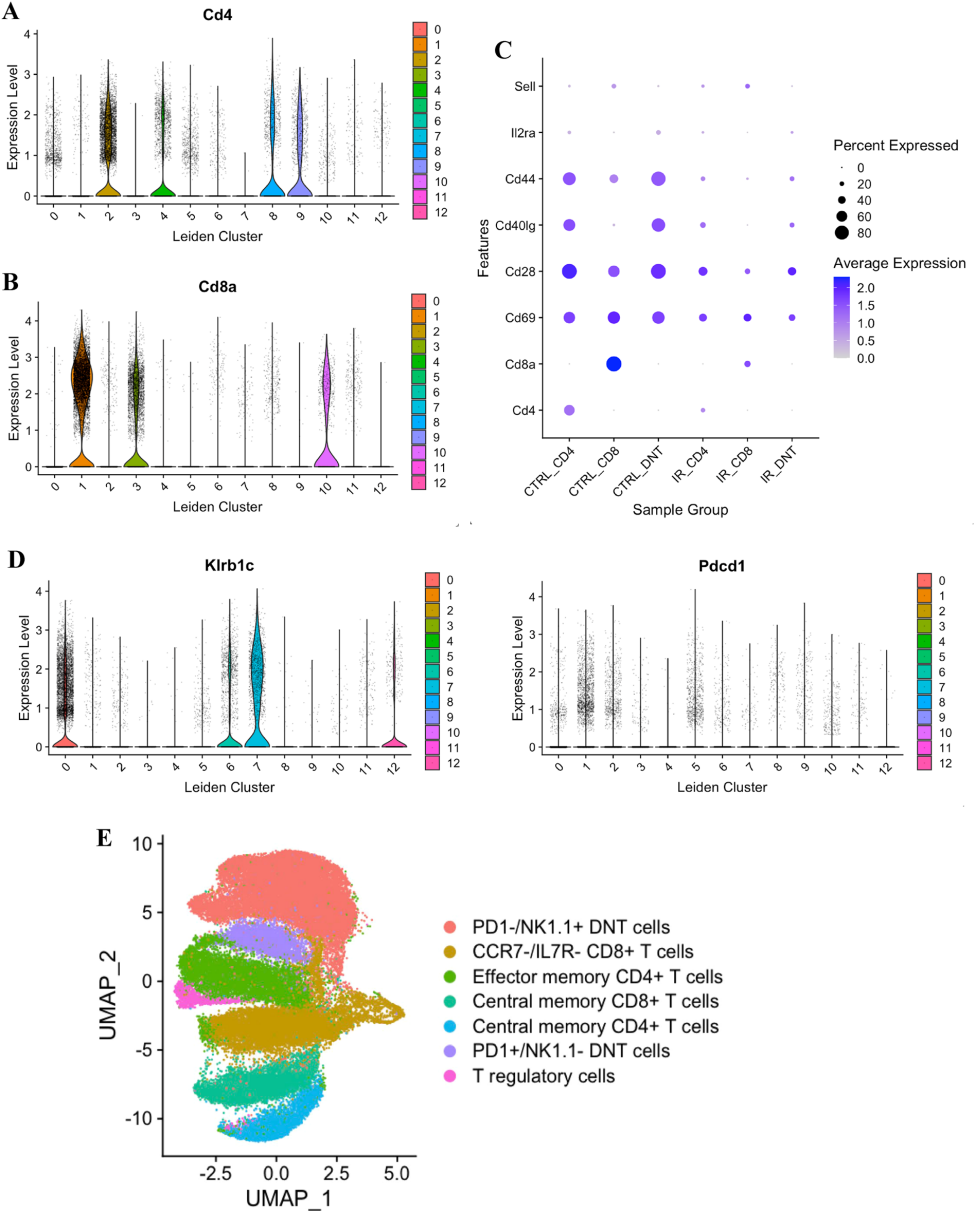
Identification of kidney T cell subtypes. (**A**) Expression of CD4^+^ and (**B**) CD8^+^ in different UMAP clusters. (**C**) Expression of T cell markers on control and ischemic CD4^+^, CD8^+^, and double negative (DN) T cells. (**D**) Identification of natural killer (NK)1.1^+^ and programmed cell death protein 1 (PD1^+^). (**E**) Final annotated UMAP for different cell types.

We identified the major transcriptional states in our data by clustering using the Leiden graph-based community detection algorithm^22^. We identified 15 clusters which were annotated given expression levels of hematopoietic lineage marker genes and differential gene expression analysis. We first identified T cell clusters (CD45^+^ CD2^+^ CD3^+^) and excluded non-T cells: three out of 15 clusters did not express T cell marker *Cd3* or T/ natural killer (NK) cell marker *Cd2* and instead expressed several myeloid lineage markers. Therefore, those clusters (*n* = 587 cells) were thought to represent contaminants and were excluded from future analysis. Visualization of sample group distribution on the uniform manifold approximation and projection (UMAP) embedding demonstrates the clear partitioning of CD4^+^, CD8^+^, and DN T cells from normal and ischemic samples (Fig. 1B). We repeated our analysis on the remaining cells to obtain 12 final clusters (Fig. 1C). Next, we performed cluster annotation by analyzing expression levels of known T cell subtype marker genes. We used different markers as proxies to identify the expression of TCR alpha, beta, delta, and gamma constant subunits. As expected, we observed elevated expression of α/ß-TCR marker genes (*Trac* and *Trbc1*) and scant expression of δγ-TCR marker genes (*Trdc, Trdv1, Trgv2,* and *Trgj2*) in all clusters apart from sparse low-level expression of *Trgv2* in clusters 0, 5 and 7, indicating that clusters 1–12 are TCRαβ^+^ (Fig. 1D). Next, we evaluated expression levels of *Cd4* and *Cd8a* to confirm the identity of flow-sorted cells and annotate CD4^+^ and CD8^+^ clusters. While *Cd4* was expressed by cells in clusters 2, 4, 8, and 9 (Fig. 2A), *Cd8a* was expressed by cells in clusters 1, 3, and 10 with scant expression in clusters 8 and 11 (Fig. 2B). Overall, expression of CD4^+^ and CD8^+^ was as expected within cells of each sample group (Fig. 2C). Next, we identified that the CD4^+^, CD8^+^, and DN T cells were expressing high levels of CD69, CD28, CD44, CD25 (IL2RA) and low levels of CD62L markers (SELL, an L-selectin) (Fig. 2C). We previously identified two major subsets of kidney DN T cells, programmed cell death protein 1 (PD-1^+^) and NK1.1^+^, both in mice and humans^8,10,23^. Using *Klrb1c* and *Pdcd1* expression markers, we identified NK1.1^+^ highly expressing in clusters 0, 6, 7, and 12, while clusters 1 and 5 contain PD1^+^ cells (Fig. 2D). Additionally, we used *CD3, Ncam1,* killer cell lectin like receptor B member 1 (*Klrb1*) *B, Klrb1c, CD24*, and *CD44* markers to identify NKT cells (Supplementary Fig. 1).

To check the expression of naïve and central memory T cells, we used CCR7, CD27, and CD62L/SELL markers. No naïve T cell clusters (CCR7+IL7R−) were identified in our data, however two central memory T cell clusters were identified including clusters 3 and 4 that were enriched in *Ccr7* and *Il7r* expression (Supplementary Fig. 2A). To annotate effector memory T cell clusters, we identified CCR7- CD37- CD62L/SELL lo/variable, IL7R+ clusters as clusters 0, 2, 7, 8, and 12 (Supplementary Fig. 2A). Finally, we annotated cluster 9 as the CD4+CD25/Il2ra+FOXP3+ regulatory T cell (Supplementary Fig. 2B). The final annotated UMAP for different cell types is provided (Fig. 2E).

### Differential gene expression in DN T cells

**Figure 3:**
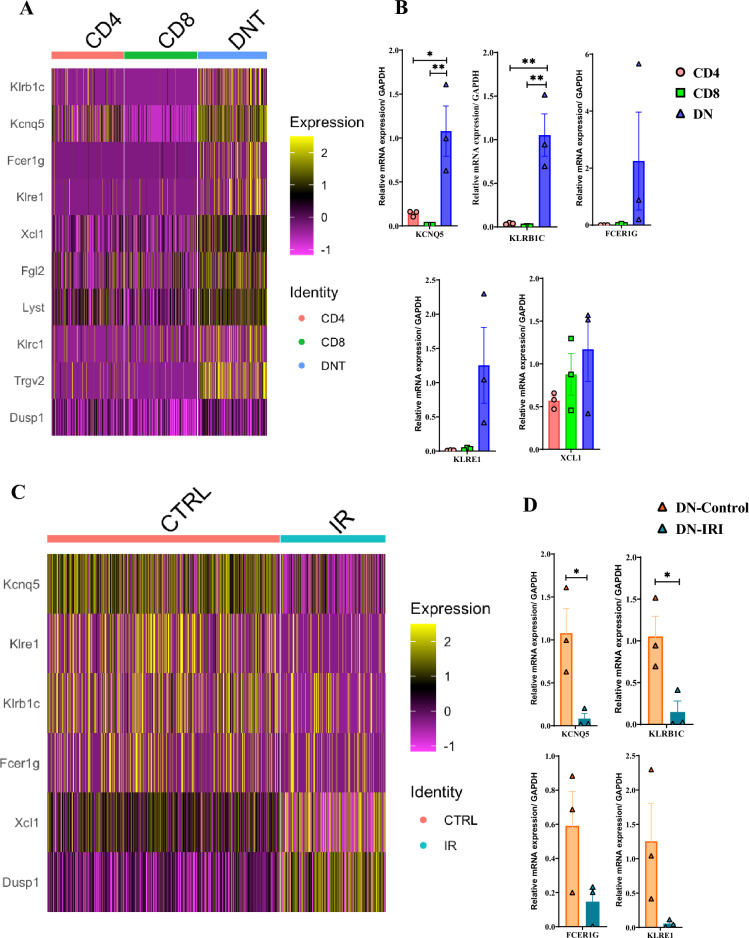
Comparison of gene expressions between the cell types. (**A**) Heat map of top differentially expressed genes for control CD4 vs CD8 vs DNT. (**B**) Validation of gene expression in control CD4, CD8, and DN T cells with a significantly higher expression in potassium voltage-gated channel subfamily Q member 5 (*Kcnq5*) and killer cell lectin like receptor B member 1 C (*Klrb1c)* DN T cells compared with CD4^+^ and CD8^+^ T cells. (**C**) Heat map of top differentially expressed genes for control DN vs ischemic DN T cells. (**D**) Validation of gene expression in control DN and ischemic DN T cells with significantly higher expression of *Kcnq5* and *Klrb1c* genes in control DN T cells compared to the ischemic DN T cells. Data in C displayed as mean ± SEM.; multiple comparisons by one-way ANOVA *P < 0.05 and **P < 0.01, data in (**D**) displayed as mean ± SEM.; multiple unpaired t test *P < 0.05.

We next sought to identify genes whose expression is distinct to kidney DN T cells compared to kidney CD4^+^ and CD8^+^. In T cells from our control group, we found higher expression of *Klrb1c* (logFC = 0.949, p_adjust_ = 0), potassium voltage-gated channel subfamily Q member 5 (*Kcnq5*, logFC = 1.18, p_adjust_ = 0), *Fcer1g* (logFC = 0.770, p_adjust_ = 0), killer cell lectin-like receptor family E member 1 (*Klre1,* logFC = 0.860, p_adjust_ = 0), X-C Motif Chemokine Ligand 1 (*Xcl1,* logFC = 0.671, p_adjust_ = 0), Fibrinogen Like 2 (*Fgl2,* logFC = 0.926, p_adjust_ = 0), Lysosomal trafficking regulator (*Lyst,* logFC = 0.773, p_adjust_ = 0), Killer Cell Lectin Like Receptor C1 (*Klrc1*, logFC = 0.626, p_adjust_ = 0), T Cell Receptor Gamma Variable 2 (*Trgv2,* logFC = 1.08, p_adjust_ = 0)*,* and dual specificity phosphatase 1 (*Dusp1,* logFC = 0.325, p_adjust_ = 8.32*10^–247^) genes in DN T cells compared with CD4^+^ and CD8^+^ T cells (Fig. 3A). Next, we validated the mRNA expression of 5 genes including *Klrb1c*, *Kcnq5*, *Fcer1g*, *Klre1*, and *Xcl1* using qPCR (Fig. 3B). Out of these 5 genes, a significantly higher expression was found in *Kcnq5* and *Klrb1c* DN T cells compared with CD4^+^ (*P* = 0.017 and *P* = 0.006 respectively) and CD8^+^ (*P* = 0.009 and *P* = 0.004 respectively) T cells. We observed a tendency for increased expression of *Fcer1g*, *Klre1*, and *Xcl1* genes in DN T cells compared with CD4^+^ and CD8^+^ T cells, although this difference did not reach statistical significance. Furthermore, we identified genes expressed in both control and ischemic DN T cells (Supplementary Fig. 3) and focused on differentially expressed genes *Kcnq5*, *Klre1*, *Klrb1c*, *Fcer1g*, *Xcl1*, and *Dusp1,* where expression of *Dusp1* is enriched in IR DN T cells (logFC = 1.02, p_adjust_ = 0) and *Fcer1g, Kcnq5, Xcl1,* and *Klrb1c* are enriched in control DN T cells (logFC = 0.087, 0.976, 0.188, and 0.358, p_adjust_ = 1.0, 0.0, 7.50*10^–13^, and 1.66*10^–34^, respectively) (Fig. 3C). Next, we investigated expression levels of *Kcnq5* and *Klrb1c* genes in control DN T cells compared with the ischemic DN T cells (*P* = 0.027 and *P* = 0.03 respectively) using qPCR. A trend in higher expression of *Klre1* and *Fcer1g* genes in control DN T cells compared with the ischemic DN T cells was observed, however, it was not significant (Fig. 3D). We then mined the NIH kidney precision medicine project (KPMP) kidney tissue atlas data base (http://www.kpmp.org) and confirmed the expression of *Kcnq5*, *Klrb1*, and *Fcer1g* genes in human kidney (Supplementary Fig. 4A). In addition, we checked expression of *Fcer1g* in human kidney using http://www.proteinatlas.org and we found this gene is mainly expressed in macrophages, monocytes, Hoffbauer cells, Langerhans cells, macrophages, Kupffer cells, granulocytes, dendritic cells, T cells, and B cells. Out of T cells we found higher expression of this gene in DN T cells compared with CD4 and CD8 T cells (Supplementary Fig. 4B).

### Spatial organization of putative DN T cells in normal and ischemic kidney

**Figure 4:**
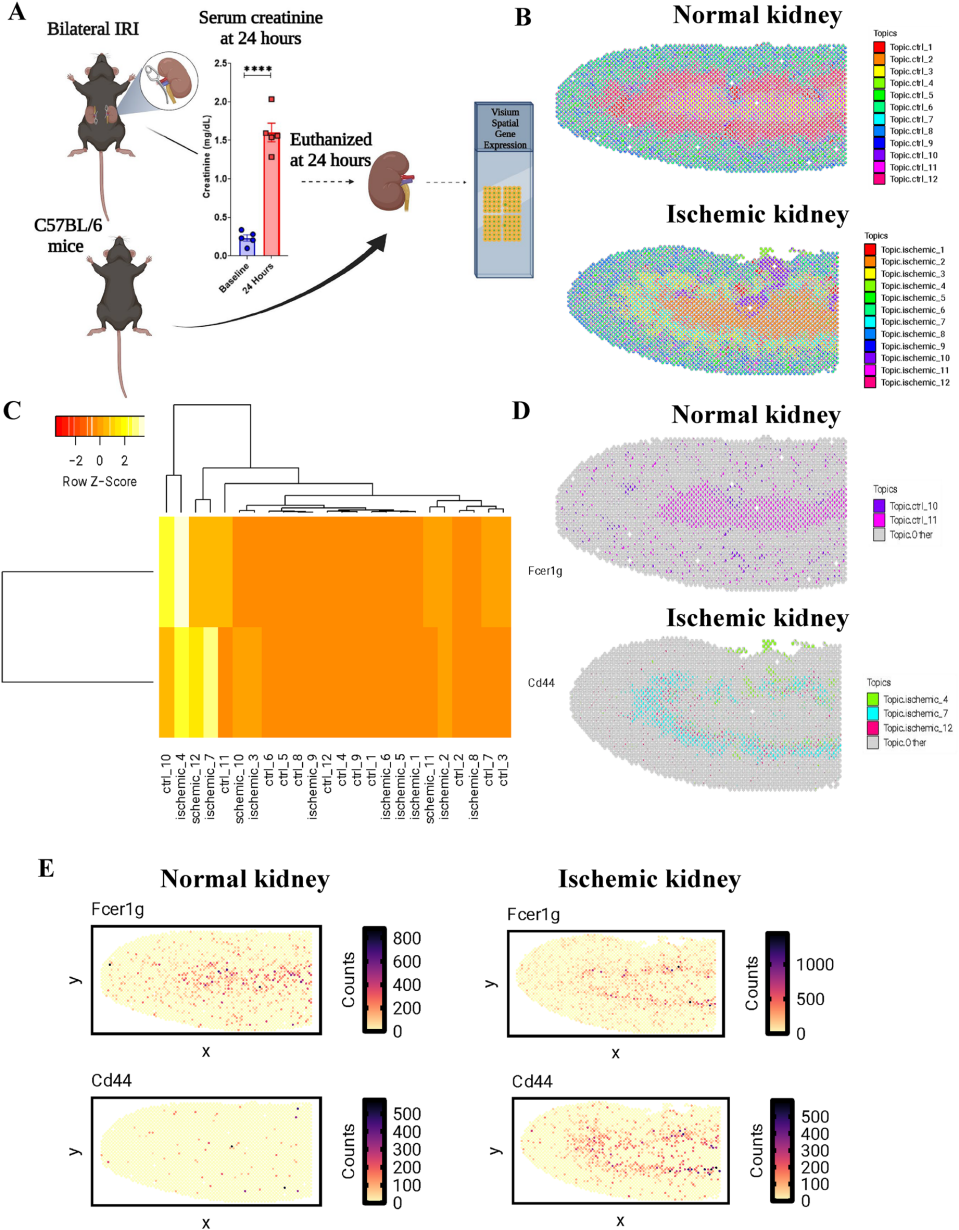
Spatial organization of putative DNT cells in normal and ischemic injury kidney samples. (**A**). Schematic showing the workflow of generating each 10X Visium spatial dataset. (**B**). Deconvolved cell type spot proportions for Visium data of normal (top) or ischemic (bottom) kidney tissue sections represented as pie charts. (**C**). Heatmap of scaled gene expression for Fc Epsilon Receptor Ig (*Fcer1g*) and *Cd44* across the deconvolved cell types. (**D**). Spot proportions of normal kidney cell type 10 and 11 (top) and ischemic injury cell type 4, 7, and 12 (bottom). (**E**). Counts per million normalized gene counts in each spot of the normal and ischemic kidney Visium datasets for *Fcer1g* and *Cd44*.

To assess the spatial organization of DN T cells in normal and ischemic injury mouse kidney, we generated ST datasets from 4 normal and 4 ischemic injury kidney sections using the 10X Genomics Visium platform (Fig. 4A). These generated ST datasets provide the average transcriptional profile for multi-cellular spots tiled across our kidney sections, where each spot may be mixtures of multiple cell-types, necessitating deconvolution analysis to recover cell-type specific organization (Fig. 4B). As our scRNA-seq dataset was limited to only a few selected cell types, we utilized a reference-free approach to deconvolve putative cell type transcriptional profiles and proportional information across the spots. In total, we deconvolved 24 cell types between the normal and ischemic condition kidney samples (12 for each condition) (Fig. 4C). To determine if any of these cell types were representative of a DN T cell type, we compared the scaled expression levels of genes across the deconvolved cell type transcriptional profiles for immune markers with sufficient capture efficiency across the ST datasets, such as *Cd44*, a marker for a major subset of kidney DN T cells, and *Fcer1g*. Other DN T cell type associated genes identified in the scRNA-seq data were not detectable or captured at sufficient levels across spots in the ST datasets (Supplementary Figs. 5, 6). We found that deconvolved cell type 10 and 11 in the normal kidney and deconvolved cell type 4, 7 and 12 in the ischemic kidney were both enriched for these genes with respect to the other cell types (Fig. 4C). The highest deconvolved proportions of these two cell types in the normal or ischemic kidney samples are localized in the pelvic region or around outer medullary regions, respectively (Fig. 4D, Supplementary Fig. 7). Moreover, the spatial expression patterns of *Fcer1g*, and *Cd44* also correspond spatially to the deconvolved proportions of these cell types in normal and ischemic injury kidney (Fig. 4E). Notably, the deconvolved proportions of these cell types also did not correspond to spatial expression patterns of *Cd4*, *Cd8a*, and *Cd8b1*, further supporting this notion that they may be representative of DN T cells in each kidney condition (Supplementary Figs. 8, 9).

## Discussion

T cells participate in experimental AKI and repair, but the molecular mechanisms of individual cell types, particularly the more recently identified DN T cells, is not known. In this study, we performed scRNA-seq of kidney T cells and spatial transcriptomics of normal and post ischemic mouse kidney to better understand the kidney DN T cell single cell transcriptional landscape and spatial transcriptomic responses. We identified and validated the differential expression of genes associated with DN T cells and compared them with that of CD4^+^ and CD8^+^ cells in murine normal and post ischemic kidneys. We integrated bioinformatic and deconvolution approaches in spatial transcriptomics. We propose that the *Fcer1g* gene as a putative DN T cell marker in both the normal and ischemic kidney.

We found that the expression of *Kcnq5*, encoding the Kv7.5 potassium channel, is significantly higher in normal kidney DN T cells compared with normal CD4^+^, CD8^+^, and ischemic kidney DN T cells. Little is known about the function of this gene in normal and ischemic kidney but it may be involved in DN T cells homeostasis and function, possibly serving a protective role in normal kidney DN T cells. Voltage-gated “Kv” channels encoded by Kcnq1–5 (Kv7.1–Kv7.5) are key regulators of the smooth muscle resting membrane potential and contractility in animal and human arteries^24^. Vascular smooth muscle expresses mainly Kv7.1, Kv7.4 and Kv7.5 channels. In a previous study, renal segmental arteries of Wistar rats were examined using RT-PCR as well as isometric myography and intact kidneys explored using constant flow perfusion. Kv7.1 channel activation improved renal perfusion without altering vasoconstrictor- or vasodilator-evoked regulation suggesting that these channels may serve as targets for renoprotection^25^.

We found a higher expression of *Klrb1c* gene in DN T cells. PD-1^+^ and NK1.1^+^ are two major subsets of kidney DN T cells^8^. *Klrb1c* encodes NK1.1 which is a transmembrane subunit of the co-inhibitory killer cell lectin-like receptor subfamily B. Previously, *Klrb1c* was identified as a gene associated with anti-tumor immunity when whole transcriptome profiling was performed using RNA-seq to dissect the molecular mechanism underlying resistance to anti-PD-1 immunotherapy in MC38 tumors^26^.

We also found a downregulation of the *Klre1* gene in ischemic kidney DN T cells. A study using scRNA-seq to identify the role of ILC subgroups in the tumor immunity of colorectal cancer (CRC) revealed the expression of inhibitory receptors including *Klre1* at a late stage of CRC^27^.

*Xcl1*, also referred to as lymphotactin, is a chemokine (C motif) ligand that is synthesized by CD4^+^ and CD8^+^ T cells, as well as NK cells. Among these immune cells, activated CD8^+^ T cells are the primary source of *Xcl1* production. In a prior study, in the context of Mycobacterium tuberculosis infection, activated CD8^+^ T cells within the lungs were responsible for the production of *Xcl1*. This chemokine also played an essential role in modulating the production of interferon-γ by CD4^+^ T cells^28^. However, our data revealed *Xcl1* gene is expressing with higher trend in control DN T cells compared with CD4^+^ and CD8^+^ T cells that could suggest the potential context-dependent roles of this gene in immune responses within different tissues or conditions.

*Fcer1g* was first identified as a Fc Epsilon receptor Ig and is now known to have broad roles as an adapter protein that transduces activation signals from various immunoreceptors. Of the five genes we targeted, we were able to detect *Fcer1g* gene in control and ischemic kidneys in both single cell and spatial transcriptomic data. Higher expression of *Fcer1g* could be correlated with the protective effect of DN T cells in the kidney. According to our spatial transcriptomic data, *Fcer1g* is highly expressed in the outer medullary region, which is the major region of the kidney that becomes injured in AKI. Therefore, to effectively visualize and study the outer medullary region of the kidney, a sagittal sectioning technique was employed, bisecting the kidney through its middle axis.

Thus, the observed higher *Fcer1g* expression needs to be considered in trying to understand the protective role of DN T cells in AKI. A recent study suggests a subpopulation of T-cells with *Fcer1g* may have a unique role in cancer cell sensing^29^. Previous studies identified and validated high expression of *Fcer1g* gene in association with clear cell renal cell carcinoma, the most common histological subtype of kidney cancer, with high morbidity and mortality rates worldwide^30^, progression and prognosis^31,32^. However, in another study the expression level of *Fcer1g* negatively correlated with myeloma progression, and high *Fcer1g* expression was suggested as a favorable biomarker in multiple myeloma patients^33^. A significant positive correlation was also identified between expression of *Fcer1g* and the infiltration levels immune cells using the immune infiltration analysis, implicating the role of this gene in regulating tumor immunology in endometrial cancer^34^. While human atlas protein data base displays highly expression of this gene in other kidney cell type, our data demonstrated within the T cell subset, DN T cells exhibited significantly higher expression levels of this gene in comparison to CD4^+^ and CD8^+^ T cell populations. This observation suggests a potential role for *Fcer1g* in the regulation or function of DN T cells within the immune system.

Our study had a number of limitations. First, while flow sorting of different cell types increased the purity of cells of interest, it may have led to changes compared to traditional isolation kits. Second, while we were able to purify CD4^+^, CD8^+^, and DN T cell populations for subsequent molecular analysis via scRNA-seq, identification of these specific subtypes in the spatial transcriptomics data remained challenging. This was in part due to inherent technical and biological differences between the scRNA-seq and ST datasets that limited the utility of the scRNA-seq data as a reference to detect distinct cell types in the multi-cellular resolution spots. Nonetheless, we were able to take advantage of a reference-free approach to deconvolve cell types in the ST datasets^35^ and subsequently annotate the deconvolved cell types using marker genes associated with DN T cells identified in our scRNA-seq data. We conducted an analysis in which we compared the transcriptional responses of different types of cells that were isolated from both normal and ischemic kidneys. Specifically, we compared these cell profiles to data obtained from individual cells. We did not find clear correlations between these datasets. The single cell RNA seq data focused on three specific types of immune cells. However, the spatial transcriptomic data encompassed all cell types present in the tissue. These spatial data points, referred to as "spots," likely contained different combinations of immune cells. The method we used to break down these spots into individual cell types (known as deconvolution) seemed to capture general features related to immune cells, but these features weren't specific enough to strongly correlate with any one of the immune cell types. Considering the challenge in directly correlating the deconvolved cell types with the three known immune cell types, we opted for a different approach. We examined the transcription profiles of the deconvolved cell types and investigated whether these profiles were enriched with specific marker genes that were previously identified in the individual cell analysis, such as the gene Fcer1g (depicted in Fig. 4C). Not all marker genes derived from the scRNA-seq data were detectable in the ST data, however, key genes such as *Fcer1g* were and therefore allowed us to identify putative DN T cell signatures in both the normal and ischemic kidney samples. Studying small populations of kidney T cells with spatial transcriptomics is likely still limited by the resolution of the technology, better suited currently for much more abundant renal epithelial cells and more prevalent white cells like macrophages^18,36^.

## Conclusions and future directions

In summary, integration of scRNA-seq and ST data enabled identification of candidate renal DN T cells gene expression signatures and expression changes between normal and ischemic murine kidney conditions. These new technologies allow for novel insights into this relatively understudied kidney T cell. However, spatial transcriptomics is still a developing technology that needs more refining to study rare kidney T cells. Among the candidate genes we have identified in mice and also found to be in humans, *Fcer1g* seems particularly promising and further pathophysiologic studies will be needed to determine its role in DN T cells during AKI. The current data provides a template for future studies on resident DN T as well as CD4^+^ and CD8^+^ T cells in healthy and diseased human kidney.

## Methods

### Animals and procedure

C57BL/6J male mice were purchased from Jackson Laboratory. All mice were kept under specific pathogen-free conditions at the Animal Facility of Johns Hopkins University. Age-matched mice between the age of 7 to 8 weeks were used in the study. All experiments were performed using experimental protocols approved by the Animal Care and Use Committee of Johns Hopkins University. All methods were performed in accordance with the relevant guidelines and regulations. This study is reported in accordance with ARRIVE guidelines.

An established model of bilateral renal ischemia–reperfusion in mice was used as previously described^37^. Briefly, mice were anesthetized with an intraperitoneal injection of sodium pentobarbital (75 mg/kg). After an abdominal medial incision, both renal pedicles were dissected, and a microvascular clamp (Roboz Surgical Instrument, Gaithersburg, MD) was placed on each renal pedicle for 30 min. Animals were kept well hydrated with warm saline and at constant body temperature (37 °C). After 30 min of ischemia, the clamps were removed and wounds were sutured. The animals were allowed to recover with free access to food and water. Serum creatinine levels were determined at baseline and 24 h after renal ischemia to confirm the injury.

### Isolation of kidney mononuclear cells

Mononuclear cells from kidneys were isolated as previously described^38^. Briefly, kidneys were minced and incubated in collagenase D (5 mg/ml; Sigma-Aldrich, St. Louis, MO) solution for 30 min at 37 °C. Single-cell suspensions of kidney digestions were obtained by mechanical disruption of tissues using 70-μm strainers (BD Bioscience) and KMNC were isolated using Percoll density gradient centrifugation as previously described^38^. The numbers of viable lymphocytes in each sample were determined using trypan blue exclusion dye under a light microscope.

### Cell sorting and gating strategy

Fluorochrome-conjugated mouse antibodies (mAbs) were purchased from Invitrogen, BioLegend, or BD Pharmingen for cell sorting. T lymphocyte cell staining was performed using standard methods. Briefly, cells were stained in FACS buffer (PBS, 2% FBS, 0.1% sodium azide) and preincubated with anti-CD16/CD32 antibody for 10 min to minimize nonspecific binding through Fc-receptors. We used the following mAbs to mouse (m) and human (h) antigens: mCD45–APC- Cyanine7 (30-F11), mTCR- BV421 (H57-597), mCD8α-APC (53–6.7), mCD4- PerCP-Cyanine5.5 (GK1.5), hCD45–APC- Cyanine7 (2D1), hTCR- BV421 (IP26), hCD8α-APC (RPA-T8), h CD4-PE (RPA-T4), and live/dead fixable aqua dead cell stain kit for 405 nm excitation.

Cells were stained by incubation with the appropriate cocktails of fluorochrome-conjugated monoclonal antibodies for 30 min at 4 °C, washed, and stained with live/dead aqua followed by incubation for 30 min. Cells were then sorted with a FACS Aria II Cell Sorter (BD Biosciences). After sorting purified CD4^+^, CD8^+^, and DN T cells from normal and ischemic mice were used for single cell RNA-Seq analysis.

### cDNA synthesizes and library preparation

To capture and barcode individual cells using 10X Chromium technology, purified sorted cells were processed for high-throughput droplet encapsulation. Cell counts and viability were determined using the Cell Countess II with Trypan Blue. A maximum volume of 43.3 µL/sample was used for processing to target up to 10,000 cells. Cells were combined with RT reagents and loaded onto 10X Next GEM Chip G along with 3’ v3.1 gel bead. The NextGEM protocol was run on the 10X Chromium Controller to create GEMs, composed of a single cell, uniquely barcoded gel bead, and RT reagents. 100 µL of emulsion was retrieved from the chip and incubated (45 min at 53 °C, 5 min at 85 °C, cool to 4 °C), generating barcoded cDNA from each cell. The GEMs were broken using Recovery Agent and cDNA was cleaned, following manufacturer’s instructions using MyOne SILANE beads. cDNA was amplified for 11–12 cycles, depending on input cell number (3 min at 98 °C, 11–12 cycle: 15s at 98 °C, 20 s at 63 °C, 1 min at 72 °C; 1min at 72 °C, cool to 4 °C). Samples were cleaned using 0.6X SPRIselect beads. QC was completed using Qubit and Bioanalyzer to determine size and concentrations. 10 µL of amplified cDNA was carried into library prep. Fragmentation, end repair and A-tailing were completed (5 min at 32 °C, 30 min at 65 °C, cool to 4 °C), and samples cleaned up using double sided size selection (0.6×, 0.8×) with SPRIselect beads. Adaptor ligation (15 min at 20 °C, cool to 4 °C), 0.8× cleanup and amplification were performed, with PCR using unique i7 index sequences. Libraries underwent a final cleanup using double sided size selection (0.6×, 0.8×) with SPRIselect beads. Library QC was performed using Qubit, Bioanalyzer and KAPA library quantification qPCR kit. Libraries were sequenced on the Illumina NovaSeq 6000 using v1.5 kits, targeting 50K reads/cell, at read lengths of 28 (R1), 8 (i7), 91 (R2). Demultiplexing and FASTQ generation was completed using Illumina’s BaseSpace software.

### scRNA-Seq analysis of kidney T lymphocytes

In this study, data from scRNA-Seq was preprocessed by 10× Genomics using the default settings. CellRanger (version 6.0.1) was used for alignment of the sequence with the mm10 reference genome. Additionally, Seurat (version 4.1.1) was used to filter quality control data and perform clustering and downstream gene expression analysis in R^39,40^. We performed quality control to exclude potential doublets and low-quality libraries, excluding cells with the top 5% of total reads per capture and cells with percent mitochondrial gene counts less than 20%. Features expressed in fewer than 5 cells, mitochondrial and ribosomal features, and *Malat1* were also excluded. In order to identify highly variable genes, data was first normalized, and then log transformed and scaled. Principal component analysis was performed using the RunPCA function in Seurat, where the number of PCs to account for the majority of variation in the dataset were selected from inspection of variance ratio ‘elbow’ plots. Batch correction on computed PCs was performed in Harmony using the RunHarmony function (version 0.1.0)^21^. A nearest neighbors graph was generated using the FindNeighbors function, then Leiden clustering was performed^22^ and visualized with a UMAP embedding^41^. Marker gene expression, differential expression analysis, and gene set enrichment analysis were used to annotate clusters and to identify and exclude any contaminating non-T cell clusters from further analysis. Analysis was repeated as above on the remaining T cell clusters following removal of contaminants. Differential gene expression analysis was accomplished with the FindAllMarkers function in R, and gene set enrichment and expression analysis were performed with the Gseapy enrichr function^42^, testing for enrichment of the MSigDB Hallmark signatures^43^.

### qPCR verification

Total RNA was isolated from murine and human CD4^+^, CD8^+^, and DN sorted cells using a RNeasy Mini kit (Qiagen, Valencia, CA) and reverse transcribed using High-Capacity cDNA Reverse Transcription Kit (Applied Biosystems, Foster City, CA). Real-time PCR was performed in QuanStudio 12K Flex (Applied Biosystems, Foster City, CA) using the PowerUp SYBR Green master mix (Applied biosystems). The primer sequences for *Kcnq5*, *Fcer1g*, *Klre1*, *Xcl1*, and *Klrb1c* genes for mouse are listed in Table 1. Glyceraldehyde-3-Phosphate Dehydrogenase (*Gapdh*) served as the reference gene and the relative fold expression values were calculated using a ΔΔ cycle threshold method^44^. In this qPCR verification step, we used forty mice normal kidneys and forty-five mice ischemic kidneys to isolate CD4^+^, CD8^+^, and DN T cell populations for feature gene verification.

**Table 1:** Mouse primers used in study.

	Primer sequence (5′ to 3′)
Fcer1g-F	5′CTGTCTACACGGGCCTGAAC3′
Fcer1g-R	5′AAAGAATGCAGCCAAGCACG3′
Kcnq5-F	5′GTCGGCGCAACGTCAAGTA3′
Kcnq5-R	5′AACCAAACACAAGGAGAAAAACG3′
Klrb1c-F	5′GACACAGCAAGTATCTACCTCGG3′
Klrb1c-R	5′TCAGAGCCAACCTGTGTGAACG3′
Klre1-F	5′AACACAGCAGAAGACATCAGTGG3′
Klre1-R	5′CTACAGCCATCAGGAGAAGGCA3′
Xcl1-F	5′TTTGTCACCAAACGAGGACTAAA3′
Xcl1-R	5′CCAGTCAGGGTTATCGCTGTG3′

### Spatial transcriptomics

To profile the spatial transcriptome of T lymphocytes in ischemic kidney, bilateral renal ischemia–reperfusion model was performed in mice as described above. 24 h after ischemia, mice were anesthetized with intraperitoneal ketamine (130 mg/kg) and xylazine (7 mg/kg). Immediately after dissection and de-capsulation, kidneys were snap-frozen in liquid nitrogen and embedded in optimal cutting temperature (OCT) at – 80 °C. We used 2 Visium slides, totaling 8 capture areas. We profiled 2 biological replicates (i.e. 2 mice) per condition, and for each biological replicate we performed 2 technical replicates. 10 µm thick sections were cut on a cryostat at – 15 °C. Spatial transcriptomics was performed using 10X Visium Spatial Gene Expression Slide and Reagent Kit (Cat #1000187) according to manufacturer’s instructions (Protocol CG000239 Rev A), with the following sample-specific parameters: Tissue sections were permeabilized for 12 min at 37 °C. cDNA was amplified using 14 cycles based on Cq values obtained using qPCR. Based on post-amplification cDNA yield, final libraries were amplified using 13 cycles during sample index PCR. Each sample was indexed with a unique well from the Dual Index Kit TT Set A (Cat #1000215). The libraries were sequenced on an Illumina NovaSeq with an SP200 flowcell, 4 samples per lane, in paired-end mode.

### Deconvolution of cell types in normal and ischemic kidney tissues with STdeconvolve

#### Preprocessing

All eight 10X Visium datasets from either normal or ischemic injury kidney tissue samples were initially combined into a single dataset with 22,749 total spots and 32,285 total genes. Spots with poor capture efficiency defined as those with less than 100 total gene counts were removed. Genes that were counted less than 100 times across all spots or detected in less than 100 spots were removed. Additionally, all mitochondrial genes were removed. This resulted in a preprocessed dataset of 22,749 spots and 14,674 genes.

#### Feature selection and deconvolution

Starting from the preprocessed dataset, each individual sample was extracted, and feature selection was performed. Briefly, genes detected in more than 5% but less than 100% of pixels were removed. Genes that were significantly overdispersed were identified using a general additive model with a basis of 5 and an adjusted p-value cutoff of 0.05, and the top 1000 most significant overdispersed genes were retained. After, the union of overdispersed genes for each individual sample was taken resulting in a final set of 2327 overdispersed genes, which was used for subsequent deconvolution. The four normal and the four ischemic kidney samples were combined into two separate spatial datasets for reference-free deconvolution with STdeconvolve^34^ (version 1.0.0), which resulted in a normal condition dataset of 2327 genes and 11,779 spots, and an ischemic condition dataset with 2327 genes and 10,970 spots. LDA models were trained on each combined dataset using a range of Ks from 6 to 26 and in each case, selected the model with K = 12, which was the highest K that also minimized both perplexity and the number of deconvolved cell types with mean spot proportions less than 0.05. For each deconvolved cell type, its deconvolved transcriptional profile with respect to the 2327 over dispersed genes, and its predicted spot level proportions, were obtained.

#### nSpatial gene expression

Starting from the original 10X Visium counts matrix of 22,749 total spots and 32,285 total genes, counts per million depth normalization was performed on each spot.

### Statistical analysis

In this study, comparisons between multiple groups were performed by a one-way ANOVA test followed by the Tukey comparison test. Unpaired t tests were used for comparison of repeated measures in the same group. Statistical analysis was performed using Prism 9, GraphPad Software, and significance was determined as *P* < 0.05. To identify genes differentially expressed in the scRNAseq data, the ‘LogFoldChange’ and ‘'FindMarkers' Seurat functions were applied to compute log fold change values and adjusted p values, respectively; p values were computed with the Student’s t test and p value adjustment performed with Bonferroni correction. Heat maps were constructed with the ‘DoHeatmap’ function in Seurat to display the top differentially expressed genes between sample groups of interest, where genes were ranked by log fold change (Fig. 3A) and met a significance threshold of p_adjusted_ < 0.05.

### Supplementary Information

Supplementary Figures.

## Data Availability

Data generated in this single cell and spatial transcriptomics analysis of kidney double negative T lymphocytes in normal and ischemic mouse kidneys has been deposited at the Gene Expression Omnibus (GEO), a data repository of the National Institutes of Health (NIH). Reviewers and editors can access the data by using the link and the access token provided below. https://www.ncbi.nlm.nih.gov/geo/query/acc.cgi?acc=GSE233513. Access token: qlqxsmqchbelfel.
